# *Arabidopsis thaliana* Cuticle Composition Contributes to Differential Defense Response to *Botrytis cinerea*

**DOI:** 10.3389/fpls.2021.738949

**Published:** 2021-11-05

**Authors:** Wendy Aragón, Damien Formey, Norma Yaniri Aviles-Baltazar, Martha Torres, Mario Serrano

**Affiliations:** ^1^Centro de Ciencias Genómicas, Universidad Nacional Autónoma de México, Cuernavaca, Mexico; ^2^Programa de Doctorado en Ciencias Biomédicas, Centro de Ciencias Genómicas, Universidad Nacional Autónoma de México, Cuernavaca, Mexico

**Keywords:** cuticle, cuticular mutants, *B. cinerea*, permeability, ROS, cell wall

## Abstract

The chemical composition of a plant cuticle can change in response to various abiotic or biotic stresses and plays essential functions in disease resistance responses. *Arabidopsis thaliana* mutants altered in cutin content are resistant to *Botrytis cinerea*, presumably because of increased cuticular water and solute permeability, allowing for faster induction of defense responses. Within this context, our knowledge of wax mutants is limited against this pathogen. We tested the contribution of cuticular components to immunity to *B. cinerea* using mutants altered in either cutin or wax alone, or in both cutin and wax contents. We found that even all the tested mutants showed increased permeability and reactive oxygen species (ROS) accumulation in comparison with wild-type plants and that only cutin mutants showed resistance. To elucidate the early molecular mechanisms underlying cuticle-related immunity, we performed a transcriptomic analysis. A set of upregulated genes involved in cell wall integrity and accumulation of ROS were shared by the cutin mutants *bdg*, *lacs2-3*, and *eca2*, but not by the wax mutants *cer1-4* and *cer3-6*. Interestingly, these genes have recently been shown to be required in *B. cinerea* resistance. In contrast, we found the induction of genes involved in abiotic stress shared by the two wax mutants. Our study reveals new insight that the faster recognition of a pathogen by changes in cuticular permeability is not enough to induce resistance to *B. cinerea*, as has previously been hypothesized. In addition, our data suggest that mutants with resistant phenotype can activate other defense pathways, different from those canonical immune ones.

## Introduction

A cuticle is a hydrophobic structure that covers the surface of the epidermal cells of the aerial parts of plants, such as leaves, stems, flowers, seeds, and fruits; and represents one of the evolutionary adaptations that has allowed plants to counteract the adverse effects produced by biotic and abiotic factors (Jeffree, 2006; Jetter et al., 2006; Riederer, 2006; Nawrath et al., 2013). The structure and chemical composition of a cuticle vary widely among different plant species, and even between organs and stages of its development (Jeffree, 2006; Nawrath et al., 2013; Ingram and Nawrath, 2017). Despite this variability, all cuticles are mainly made up of two types of lipid compounds: cutin and waxes. Cutin is a polymer layer formed by a network of esterified ω-hydroxylated fatty acids *via* intermolecular ester bonds, leading to a three-dimensional structure that is produced and secreted by epidermal cells. Waxes comprise a mixture of very long-chain fatty acids (VLCFAs, 24–36 carbon atoms) and their derivatives, including alkanes, alcohols, and aldehydes, together with secondary metabolites, such as flavonoids and triterpenoids (Li-Beisson et al., 2013; Nawrath et al., 2013; Fernández et al., 2016).

Genetic approaches that use mutagenized populations of *Arabidopsis thaliana* (Bernard and Joubès, 2013; Yeats and Rose, 2013; Borisjuk et al., 2014; Domínguez et al., 2015; Fich et al., 2016), tomato, and maize (Isaacson et al., 2009; Javelle et al., 2010; Girard et al., 2012) have allowed for the identification of many key enzymes involved in cuticle biosynthesis and deposition. Some of these mutants, such as the cutin mutants *bodyguard* (*bdg)*, *lacs2*, *lacerata* (*lcr*/*cyp86a8), cyp86a2*/*att1, abcg32*/*pec1*, and *myb96* (Wellesen et al., 2001; Schnurr et al., 2004; Xiao et al., 2004; Kurdyukov et al., 2006; Seo and Park, 2010; Benikhlef et al., 2013; Fabre et al., 2016; Zhao et al., 2019), and the wax mutants *fiddlehead* (*fdh*/*kcs10*)*, cer1*, *cer3*/*wax2*, and *dewax* (Yephremov et al., 1999; Chen et al., 2003; Kurata et al., 2003; Rowland et al., 2007; Voisin et al., 2009; Sakuradani et al., 2013; Go et al., 2014; Liu et al., 2020) show a strong reduction in cutin and wax contents. Despite the loss of its cuticular structure, which might be thought to be detrimental to the plant, these mutants can accumulate significantly either more cutin monomers or more wax components when the other is reduced relative to wild type as a compensatory mechanism to maintain the integrity of cuticle (Voisin et al., 2009; Nawrath et al., 2013; Serrano et al., 2014).

To date, studies have identified the role of cuticular components during interaction with pathogens, showing that a cuticle is a physical and chemical barrier and that its components may act as signaling and defense molecules for both fungi and plants (Serrano et al., 2014; Aragón et al., 2017; Ziv et al., 2018). For instance, cutin monomers induce the germination of *Magnaporthe grisea* during the infection process of rice (*Oryza sativa*) (Gilbert et al., 1996), appressorium formation of the powdery mildew *Erysiphe graminis*in barley (*Hordeum vulgare)* (Francis et al., 1996), and induction of a protein kinase-mediated pathway required for the pathogenic development of *Colletotrichum trifolii* (Dickman et al., 2003). Besides the cutin monomers, specific wax components, such as very-long-chain (VLC) aldehydes, induce the pre-penetration process of *Blumeria graminis* both *in vitro* (Hansjakob et al., 2010) and *in planta* (Hansjakob et al., 2011). Additionally, other wax components, such as VLC primary alcohols of avocado (*Persea americana*), induce germination and appressorium formation of *Colletotrichum gloeosporioides* (Podila et al., 1993). In contrast, plants might perceive cutin monomers released by the action of fungal cutinase as elicitors. This hypothesis was evaluated when rice and barley plants were treated with synthetic cutin monomers (C18 fatty acids) and showed resistance to *E. graminis* and *M. grisea*, respectively (Schweizer et al., 1996). In the same way, cucumber seedlings respond to hydrolyzates of cutin by producing H_2_O_2_, which has been associated with early defense responses against pathogens (Fauth et al., 1998).

Cuticular mutants and transgenic lines have contributed to the advancement of our knowledge of how defects in cuticle structure might lead to immunity of plants upon the attack by pathogenic fungi. Fungal cutinase-expressing (CUTE) plants and *A. thaliana* cutin mutants with an altered ultrastructure and increased permeability of the cuticle, such as *lacs2* (deficient in the long-chain acyl-CoA synthetase 2 enzyme that catalyzes the synthesis of intermediates in the cutin pathway and in wax biosynthesis), *bdg* (mutated in BODYGUARD, an extracellular α/β hydrolase suggested to be involved in cutin polyester assembly), and *lcr* (mutated in *CYP86A8*, which is involved in the biosynthesis of cutin pathway), displayed increased resistance to the necrotrophic fungus *Botrytis cinerea* (Sieber et al., 2000; Bessire et al., 2007; Chassot et al., 2007; Tang et al., 2007; Voisin et al., 2009). Likewise, we have recently described that a mutant with a strong reduction in both cutin and wax contents, *eca2* (expression constitutiva del gen*ATL2)*, is resistant to *B. cinerea* and to the hemibiotrophic bacterial pathogen *Pseudomonas syringae* pv tomato strain DC3000 (*Pst* DC3000), but susceptible to *Phytophthora brassicae* compared with WT plants (Blanc et al., 2018). In contrast, the mutants *lacs2,acp4*, and *myb96* with altered cutin content exhibited enhanced susceptibility against *Pst* DC3000 (Tang et al., 2007; Xia et al., 2009; Seo and Park, 2010). Besides these cutin mutants, only a few *A. thaliana* mutants with defects in wax biosynthesis or regulation have been screened for their responses to different pathogens. For instance, *cer1-1* mutants affected in *CER1* (wax biosynthetic gene fora VLC-aldehyde decarbonylase) have a significantly reduced wax load, showed susceptibility to the necrotrophic fungus *Sclerotinia sclerotiorum* (Aarts et al., 1995; Bourdenx et al., 2011), and enhanced resistance to the biotrophic fungus *Golovinomyces orontii*, whereas, in *cer3-6* and *cer3-8* mutants affected in *CER3/WAX2/YRE* (a wax biosynthetic gene for VLC-acyl-CoA reductase), the growth and reproduction of these fungi were slightly inhibited (Rowland et al., 2007; Inada and Savory, 2011). An evaluation of *in planta* bacterial growth in *cer1-1* confirmed susceptibility to bacterium *Pst* DC3000 (Xia et al., 2009; Bourdenx et al., 2011), as well as in the mutant *cer3-6* (Lee et al., 2016). The *dewax* mutant (knockout in *DEWAX* that codified to the transcription factor DEWAX, which represses cuticular wax biosynthesis) has been reported to be more susceptible to *B. cinerea* (Go et al., 2014; Ju et al., 2017). In order to explain the resistance against *B. cinerea* observed on mutants with altered cutin composition, several reports have characterized physiological changes and the induction of defense responses. These reports include analysis on cuticular water and solute permeability (hereafter referred to as cuticular permeability), the production of reactive oxygen species (ROS), expression of genes implicated in plant defense signaling pathways (Bessire et al., 2007; Chassot et al., 2007; Voisin et al., 2009; L’Haridon et al., 2011; Blanc et al., 2018), and analysis of the abscisic acid (ABA) signaling pathway (L’Haridon et al., 2011; Cui et al., 2016). Based on these reports, a model to explain the cuticle-derived resistance to *B. cinerea* was proposed. In the cutin mutants, changes in cuticular structure and permeability allow pathogen- and/or damage-associated molecular patterns (PAMPs or DAMPs), released from both the pathogen and the plant cuticle or cell wall, respectively, to be more rapidly recognized by plant pattern recognition receptors, triggering immune responses (Serrano et al., 2014). Nevertheless, we are far away from fully understanding the early mechanisms and role(s) that cuticular components might play during the plant-fungal pathogen interaction, especially against *B. cinerea*, which leads to resistance.

In this report, we characterized mutants altered in either cutin (*bdg*, *lacs2-3*) or wax (*cer1-4* and *cer3-6*) alone, or altered in both cutin and wax (*eca2*) contents during the interaction with *B. cinerea*. We determined that while all the mutants have an increased permeability, only the cutin mutants were resistant to this pathogen. Additionally, in order to identify the molecular elements that lead to this resistance or susceptibility, we performed a genome-wide transcriptional characterization before and after the challenge with the fungus. This analysis allowed us to identify a set of genes, expressed only in mutants altered in cutin content, that have recently been described as part of resistance mechanisms against *B. cinerea* (Lionetti et al., 2017; Bacete et al., 2018; Del Corpo et al., 2020). Our study allows us to understand how modification in cuticular components activates defense responses against this agronomical important phytopathogen.

## Materials and Methods

### Plant Material and Growth Conditions

*Arabidopsis thaliana* plants were grown in a greenhouse at 22 to 23°C and 60% humidity under a long day photo period (16-h light) for 4 weeks. The following plants were used: C24 ecotype as wild-type (WT) for the *eca2* mutant altered in both cutin and wax components (Salinas-Mondragón et al., 1999; Serrano and Guzmán, 2004; Blanc et al., 2018) and Columbia-0 (Col-0) as WT for mutants altered in cutin content: *bdg* (Kurdyukov et al., 2006; Voisin et al., 2009) and *lacs2-3* (CS65776 (obtained from the Arabidopsis Biological Resource Center, ABRC) (Bessire et al., 2007). The mutants altered in wax content were *cer1-4* (SALK_008544C) (Bourdenx et al., 2011) and *cer3-6* (*yre-1*) (Rowland et al., 2007), and were kindly provided by Professor Ljerka Kunst, Department of Botany, University of British Columbia, Vancouver, BC, Canada. All the selected mutants have been genetically (confirmed homozygous lines) and chemically characterized in detail (Supplementary Table 1).

### Pathogen Infection Assays

*Botrytis cinerea* strain B05.10 was cultured on potato dextrose agar (PDA, 39 g L^–1^) plates. Spores were harvested in distilled water and filtered to remove hyphae. For inoculations, spore concentration was adjusted to 5 × 10^4^ spores ml^–1^ in 1/4 -strength potato dextrose broth (PDB, 6 g L^–1^; Sigma-Aldrich, United States). For the analysis of lesion development, six fully expanded leaves per 4-week-old soil-grown plant were inoculated with a single drop of 6 μl of a spore suspension over each leaf, and at least 30 lesions were evaluated in each experiment. The inoculated plants were covered with plastic lids to maintain moisture level and transferred to a growth chamber at 22°C and a 24-h dark cycle. After 72 hpi, symptoms were evaluated. The level of resistance (disease incidence) was expressed by the percentage of plants showing disease symptoms extending beyond the inoculation site in each mutant. The developed lesions were quantified using the Image J analysis software (Fiji Is Just Image J^1^) (Schindelin et al., 2012). The experiments were repeated with at least three individual biological replicates, each with 10 technical replicates.

### Cuticular Permeability Assays

The toluidine blue staining performed was from a previously described method (Tanaka et al., 2004; Bessire et al., 2007). Rosette leaves of 4-week-old plants were detached and immersed for 2 h in 0.025% TB (Sigma-Aldrich, United States) solution in 1/4 PDB (Sigma-Aldrich, United States) and were rinsed with tap water. Photos were used to measure the stained area (mm^2^) using imageJ see text footnote 1. For staining with calcofluor white (Sigma-Aldrich, United States), the leaves were bleached in absolute ethanol overnight, incubated in 0.2 M sodium phosphate buffer (pH 9) for 1 h, and for 5 min in 0.5% calcofluor white in 0.2 M sodium phosphate buffer (pH 9). Then, the leaves were rinsed in sodium phosphate buffer to remove excess calcofluor solution, and photographed under UV light (L’Haridon et al., 2011). Chlorophyll leakage from the rosette leaves was determined by a previously described protocol (Schnurr et al., 2004). For chlorophyll measurement, the fresh weight of detached leaves of mutants and wild-type plants was measured, and they were immersed in 80% ethanol. After 1 h, 1-ml aliquots were removed, and absorbance was measured at 664 and 647 nm. The micromolar concentration of total chlorophyll per gram of fresh weight of leaves was calculated using the equation: total micromoles chlorophyll = 7.93 (A_664_) + 19.53 (A_647_) (Lolle et al., 1997; Voisin et al., 2009). The experiments were repeated with at least three biological replicates, each with six technical replicates.

### Detection of Reactive Oxygen Species

3, 3′-Diaminobenzidine and NBT staining were performed to determine the presence of hydrogen peroxide (H_2_O_2_) and superoxide (O_2_^–^), respectively. The presence of H_2_O_2_ was visualized with 3, 3′-diaminobenzidine (Sigma-Aldrich, United States) (Thordal-Christensen et al., 1997; L’Haridon et al., 2011). Detached leaves were immersed in 1 mg ml^–1^ DAB-HCl, pH 3.8, by gentle vacuum infiltration. For superoxide (O_2_^–^) staining, detached leaves were immersed for 30 min in 0.1% nitroblue tetrazolium (NBT) chloride (Sigma-Aldrich, United States) in 50 mM potassium phosphate buffer pH 7.5 (L’Haridon et al., 2011; Lehmann et al., 2015). Following incubation, the DAB and NBT staining solutions were removed and replaced with a bleaching solution (ethanol: acetic acid: = 3:1). H_2_O_2_ was visualized as a reddish-brown stain formed by the reaction of DAB with endogenous H_2_O_2_. The O_2_^–^ content was detected as a dark blue stain of a formazan compound formed as a result of NBT reacting with endogenous O^2–^. The reactive oxygen species (ROS) production in detached rosette leaves unchallenged and challenged with the pathogen was detected using 5-(6) carboxy-2′, 7′-dichlorofluorescein diacetate (DCF-DA; Sigma-Aldrich, United States). The leaves were immersed in 60 μM of DCF-DA in a standard medium (1 mM KCl, 1 mM MgCl_2_, 1 mM CaCl_2_, 5 mM 2-morpholinoethanesulfonic acid adjusted to pH 6.1 with NaOH) (L’Haridon et al., 2011; Benikhlef et al., 2013). The leaves were then observed using a Carl Zeiss Axioplan 2 epifluorescence microscope with a GFP filter set (excitation 480/40 nm, emission 527/30 nm). Microscope images were saved as TIFF files, and the accumulation of fluorescence was quantified as pixels using imageJ see text footnote 1.

### RNA Extraction, RNA Sequencing, and Analysis

For RNA-seq, rosette leaves of the Col-0, C24, *eca2*, *bdg*, *lacs2-3*, *cer1-4*, and *cer3-6* plants were inoculated by spraying the entire leaves with spore suspension (5 × 10^4^ spores ml^–1^) of *B. cinerea.* At least eight whole rosettes of each plant were collected at 6 hpi and as well under non-infected conditions. Total RNA for RNA-seq was isolated from two different biological replicates for each mutant and WT using a Spectrum^TM^ Total RNA kit (Sigma-Aldrich, United States). Total RNA concentration and purity were measured using NanoDrop^TM^ 2000 (Thermo Fisher Scientific, Inc., Waltham, MA, United States). Library construction and sequencing were performed by Beijing Genomics Institute (BGI) Americas^2^ using DNBSeq^TM^ technology. The sequencing was performed using paired end generating 100-bp size reading. The sequences are publicly available in the following link: https://dataview.ncbi.nlm.nih.gov/object/PRJNA761130?reviewer=jgq29l4gjk5tenf7e4q3755opm. Approximately 20 million reads per sample were aligned to the *A. thaliana* transcriptome (^3^ TAIR version 10) using Bowtie2 (v2.3.5) (Langmead and Salzberg, 2012). The bioinformatics data processing summary for each mutant is shown in Supplementary Table 2. We calculated gene expression levels using the RNA-seq by expectation maximization (RSEM) method (v1.3.3) (Li and Dewey, 2011). Differentially expressed genes (DEGs) were identified using the software DESeq2 in the *Integrated Differential Expression Analysis MultiEXperiment* (IDEAMEX) (Jiménez-Jacinto et al., 2019), with a FoldChange ≥ 2, and adjusted *p*-value ≤ 0.05. Additionally, the DEGs were functionally annotated with Gene Ontology (GO) terms extracted with PANTHER (v16.0) (GO term enrichment analysis) and Database for Annotation, Visualization, and Integrated Discovery (DAVID) (v6.8), by using Fisher’s exact test and correction with an FDR. Plots were created with the ggplot2 library using RStudio (v1.4.1106). To further identify DEGs common among the cutin and wax mutants, we drew Venn diagrams using the VennDiagram package in R (v4.0.3), and the webtool Venn Diagram^4^. Figures showing heatmaps were generated using the webtool Heatmapper^5^ (Babicki et al., 2016).

### Quantitative RT-PCR Analysis

Total RNA was isolated from the frozen rosette leaves of WT and the mutants infected and non-infected with *B. cinerea* (6 hpi) collected directly into liquid nitrogen and stored at −80°C. Wet weight of 100 mg was used for RNA isolation from a pool of leaves from six plants of each genotype using TRI Reagent^®^ (Sigma-Aldrich, United States), following the instructions of the manufacturer. Sample quality was assessed by using denaturing gel electrophoresis and measured using NanoDrop 1000 Spectrophotometer (Thermo Fisher Scientific, Inc., Waltham, MA, United States). A 1-μg sample of total RNA was treated with DNase I, RNAse-free (Thermo Fisher Scientific, Inc., Waltham, MA, United States), and then used as the template for cDNA synthesis with a RevertAid H Minus RevertAid First Strand cDNA Synthesis kit (Thermo Fisher Scientific, Inc., Waltham, MA, United States). Quantitative RT-PCR (RT-qPCR) reactions contained cDNA (diluted 1/40) in Maxima SYBR Green/ROX qPCR Master Mix (2×) (Thermo Fisher Scientific, Inc., Waltham, MA, United States) and 0.5 μM of specific primers. Primers for the RT-qPCR gene expression analysis have been previously described: *AtPME17* (Del Corpo et al., 2020), *AtPME41* (Choi B. et al., 2017), *RAP2.6/ERF108* (Imran et al., 2018), and *CAT3* (Zou et al., 2015). All the reactions were performed in 96-well plates using the 7300 Real-Time PCR System and 7300 System Software (Applied Biosystems, Foster City, CA, United States). PCR conditions were 95°C initial denaturations for 15 min, 40 cycles of 15 s/95°C, 30 s/60°C, and 30 s/72°C, after each run, a dissociation curve was acquired to check for amplification specificity by heating the samples from 60 to 95°C. The relative gene expression level for each sample was calculated using the comparative Ct method (Schmittgen and Livak, 2008) and normalized with the geometrical mean of two housekeeping genes, *CF150* (AT1G72150) and *ACT2* (AT3G18780) (Serrano and Guzmán, 2004; Czechowski et al., 2005). One-way ANOVA followed by Tukey comparisons was performed to evaluate the significance of the differential gene expression using the mean values from three biological replicates for each sample.

### Statistical Analysis

One-way analysis of variance, followed by Tukey’s (honestly significant difference (HSD) comparisons, was performed to determine statistical significance. GraphPad Prism8 v 8.0.1 (GraphPad Software, San Diego CA, United States) was used. Data represent the mean ± SE. Differences at *p* < *0.001* were considered significant.

## Results

### Mutants With Alteration in Cuticular Wax or Cutin Composition Confer Differential Resistance to *Botrytis cinerea*

**FIGURE 1:**
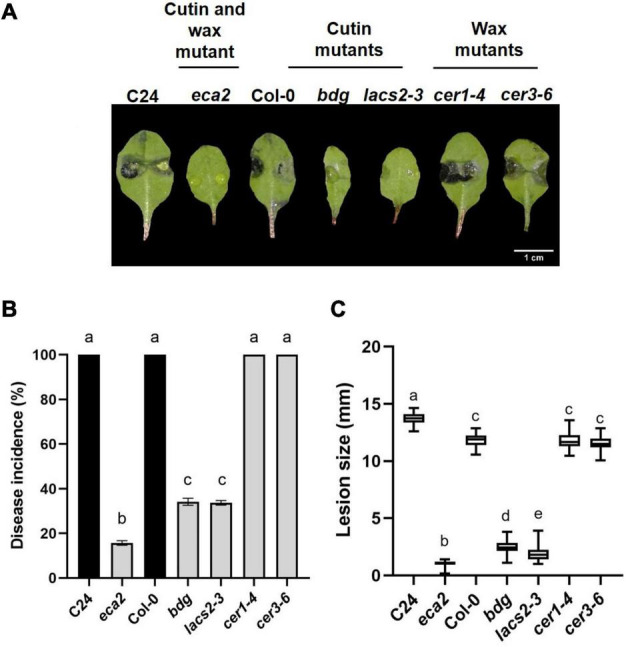
Differential phenotypic response of cuticular mutants to *B. cinerea.* Leaves of WT plants C24 and Col-0 and the cuticular mutants *eca2*, *bdg*, *lacs2-3*, *cer1-4*, and *cer3-6* were infected with *B. cinerea*. Disease symptoms on rosette leaves after 72 hpi **(A)**, disease incidence **(B)**, and lesion size **(C)** were evaluated. Three independent experiments were done (n = 60 ±SD). Different lowercase letter columns indicate significant difference, according to one-way analysis of variance (ANOVA) (*p*-value < 0.001) followed by Tukey test. Representative pictures are shown.

In recent years, a number of *Arabidopsis* mutants with defects in different steps of cutin biosynthesis, transgenic plants expressing a fungal cutinase and/or direct application of cutinase on wild type rosette leaves, have shown resistance to *B. cinerea*. These results has been interpreted as evidence of the participation of the cutin monomers in the resistance against this pathogen (Sieber et al., 2000; Chassot et al., 2007; Voisin et al., 2009). Despite the previous data from several mutants involved in the synthesis and regulation of cuticular waxes, in interaction with pathogens (Bourdenx et al., 2011; Lee et al., 2016; Ju et al., 2017), the link between wax composition/structure and the resistance or susceptibility of mutants against *B. cinerea* have not been described in detail. In addition, molecular defense mechanisms underlying the phenotypes of both the wax and cutin mutant lines mainly affected in cuticular biosynthesis are still unknown. To determine if changes in wax composition lead to resistance to this phytopathogen, we confronted the *eca2* mutant (with modified cutin and wax components), cutin mutants *lacs2-3* and *bdg*, and two mutants with reduced wax content, *cer1-4* and *cer3-6*, with *B. cinerea* and compared them with their corresponding wild-type plants. After inoculation (3 dpi), we observed that only *eca2*, *bdg*, and *lacs2-3* showed resistance to this pathogen (Figure 1A). These cutin-altered mutants exhibited less than 20 and 35% of disease incidence, respectively, while their corresponding WT plants showed an incidence of 100% (Figure 1B). However, in the *cer1-4* and *cer3-6* mutants affected only in wax composition, susceptibility similar to that of Col-0 was observed (Figures 1A,B). Additionally, the lesion average area was significantly smaller in the cutin mutants *eca2,bdg*, and *lacs2-3* compared with the wax mutants *cer1-4* and *cer3-6* (Figure 1C). One interesting observation is that under our experimental conditions, the majority of leaves from *eca*2 remained free of disease symptoms 7 dpi compared with their corresponding WT plants (C24), whereas only some of the leaves from *bdg* and *lacs2-3* remained free of disease symptoms at this time. In contrast, all the inoculated leaves of *cer1-4* and *cer3-6* already showed signs of fungal infection at 3 dpi, similar to the WT plants (data not shown). These results indicated that an altered wax composition does not correlate with resistance against the necrotrophic fungi *B. cinerea*, as previously observed in cutin mutants.

### Changes in Cutin or Wax Content Lead to Increased Leaf Permeability

**FIGURE 2:**
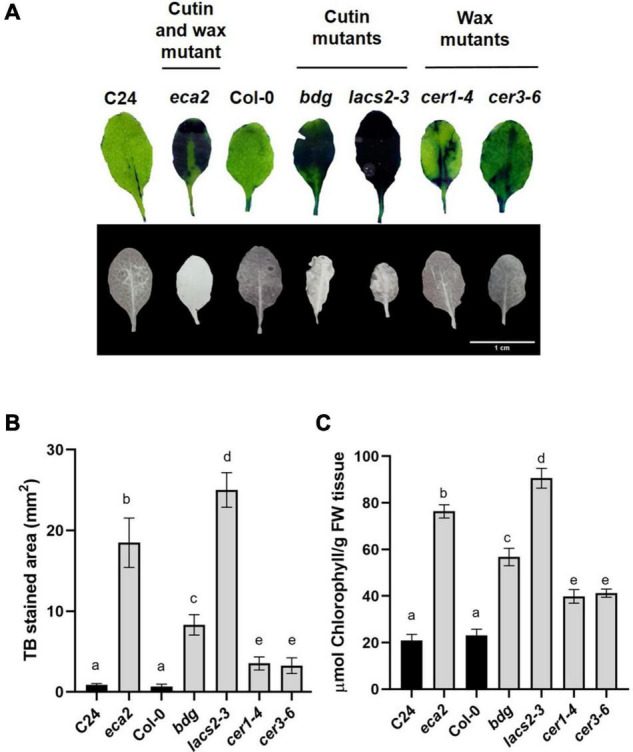
Cuticular mutants present increased leaf permeability. **(A)** Upper panel: leaves of 4-week-old WT plants and the cuticular mutants were stained with toluidine blue. Lower panel: leaves were stained with calcofluor white and viewed under UV light. **(B)** Toluidine blue-stained areas were quantified using the Fiji software. **(C)** Chlorophyll leaching 60 min after immersion of the leaves on 80% EtOH was spectrophotometrically measured as previously described (L’Haridon et al., 2011). Different lowercase letter columns indicate significant differences according to one-way analysis of variance (ANOVA) (*p*-value < 0.001) followed by Tukey’s test. Representative pictures are shown.

Previous reports on the *bdg*, *lacs2-3*, and *eca2* mutants have shown that they have a permeable cuticle (Bessire et al., 2007; Chassot et al., 2007; Voisin et al., 2009; L’Haridon et al., 2011; Blanc et al., 2018). To determine if the wax mutants present similar permeability, we assessed it by toluidine blue (TB) and calcofluor staining and measuring the increased efflux of chlorophyll, as previously described (Tanaka et al., 2004; L’Haridon et al., 2011; Cui et al., 2016). The *eca2*, *bdg*, and *lacs2-3* leaves showed the characteristic dark blue and bright patterns correlated with the TB and calcofluor stain, respectively, while *cer1-4* and *cer3-6* had weaker calcofluor staining and smaller TB-stained area compared with the cutin mutants (Figures 2A,B). Nevertheless, the quantification of the TB-stained area shows that all the mutants (such as *cer1-4* and *cer3-6*) present statistically significant increased permeability compared with their corresponding WT plants (Figure 2B). Next, we analyzed cuticular permeability by chlorophyll leaching. When non-infected rosette leaves are immersed in 80% ethanol, mutants defective either in cutin and/or wax composition lose chlorophyll more rapidly than their corresponding WT plants (Figure 2C). We observed that the cutin mutants *lacs2-3* and *eca2* had similar chlorophyll-leaching rates of approximately 4-fold more than their corresponding WT plants. The *bdg*, *cer1-4* and *cer3-6* mutants show 3- and 2-fold greater chlorophyll leaching, respectively, compared with Col-0 (Figure 2C), thus corroborating the results of the toluidine blue and calcofluor tests. Taken together, these results indicate that modification of the content of cutin and/or wax leads to a more permeable cuticle.

### Cuticle-Related Mutants Show Basal Reactive Oxygen Species Accumulation

**FIGURE 3:**
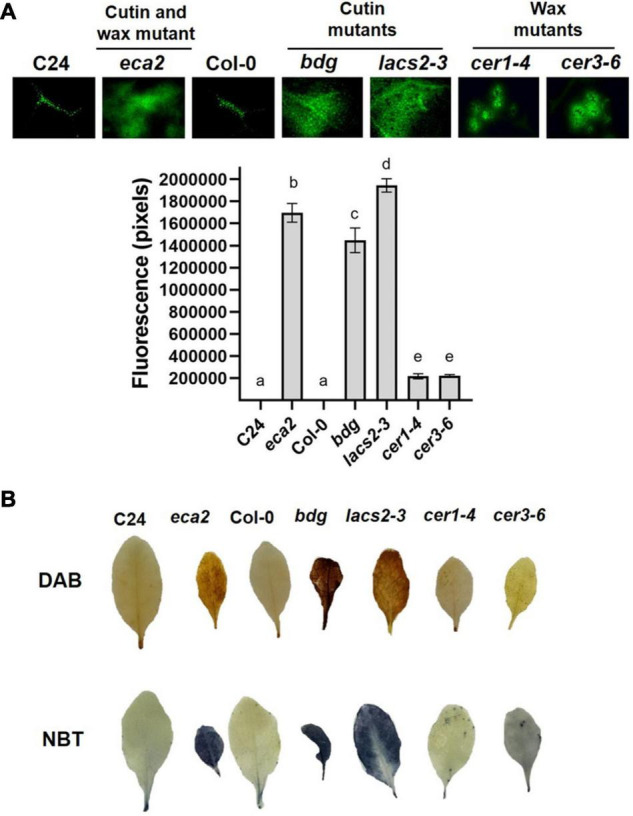
Reactive oxygen species (ROS) accumulation in the cuticular mutants and WT plants. **(A)** DCF-DA staining was observed on leaves by using epifluorescence microscopy. **(B)** 3, 3′-diaminobenzidine (DAB) and nitroblue tetrazolium (NBT) were used to detect the accumulation of hydrogen peroxide (H_2_O_2_) and superoxide (O_2_^–^), respectively. Six independent experiments were carried out with similar results (*n* = 6 ± SD). Scale bar = 100 μm. Different lowercase letter columns indicate significant differences, according to one-way analysis of variance (ANOVA) (*p*-value < 0.001) followed by Tukey’s test. Representative pictures are shown.

Previous reports on *Arabidopsis* mutants with defects in cuticle structure associated with alterations in the composition of cutin monomers showed increased reactive oxygen species (ROS) levels, even when leaves were not challenged with a pathogen (Chassot et al., 2007; L’Haridon et al., 2011; Blanc et al., 2018). To test if the production of ROS, one of the most early and rapid defense reactions to pathogen attack, is present in mutants with alterations in the composition of cuticular wax, we evaluated the accumulation of ROS in uninfected leaves of *eca2, bdg*, *lacs2-3, cer1-4*, and *cer3-6* using three different dyes: 5-(and-6)-carboxy-2,7-dichlorodihydrofluorescein diacetate (DCF-DA) that detects a broad range of oxidizing reagents (L’Haridon et al., 2011); 3, 3′-diaminobenzidine (DAB), which detects H_2_O_2_; and nitroblue tetrazolium (NBT), which detects O_2_^–^(Thordal-Christensen et al., 1997; Mengiste et al., 2003). Stronger DCF-DA fluorescence was observed in *eca2*, *bdg*, and *lacs2-3* compared with the mutants with altered wax content, *cer1-4* and *cer3-6* (Figure 3A). Nevertheless, ROS accumulation in the latter mutants was stronger than in Col-0 (Figure 3A). The DAB and NBT staining showed that coloration in the mutants with altered cutin (*eca2, bdg*, and *lacs2*) was much darker than in *cer1-4* and *cer3-6*, indicating higher ROS accumulation (Figure 3B). To further study this immune response, we analyzed ROS accumulation in leaves at 6 hpi with *B. cinerea*. We observed that all the mutants, such as *cer1-4* and *cer3-6*, showed stronger accumulation than their corresponding WT plants in all the staining methodologies (DCF-DA, DAB, and NBT) (Supplementary Figure 1). At this point, our data illustrate a possible correlation between cuticle permeability, ROS production, and *B. cinerea* resistance associated with alterations in the composition of cutin monomers, as previously described. However, although cuticle permeability and ROS accumulation are observed in the wax mutants, their susceptibility suggests that these changes might not only be associated with the resistance against *B. cinerea*, as we have previously hypothesized.

### Reduction in Cutin and Wax Contents Induced Differential Transcriptional Changes

**FIGURE 4:**
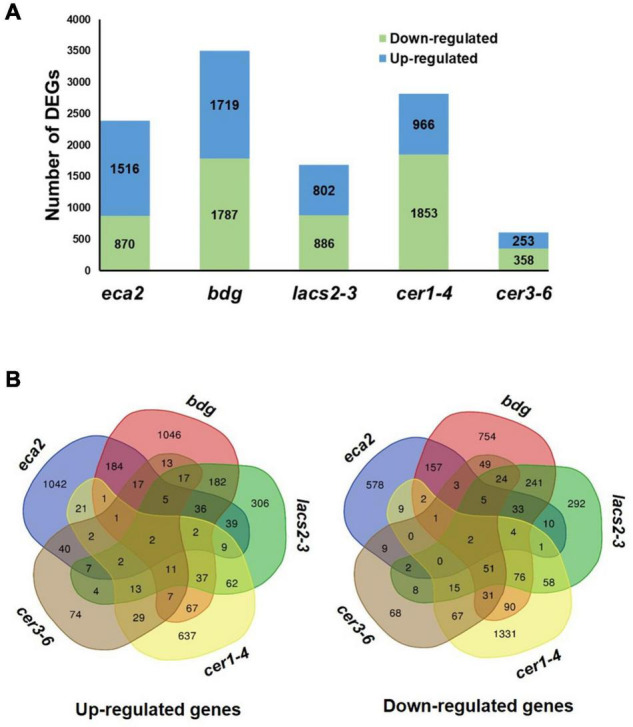
Transcriptome analysis of non-infected cuticular mutants. **(A)** Number of differentially expressed genes (DEGs) (Log2FC ≥ 1 or ≤ –1 with an adjusted *p*-value ≤ 0.05) identified in the cutin and wax mutants. **(B)** Venn diagrams show the overlap of upregulated and downregulated genes among the cutin or wax mutants.

To investigate the molecular basis that might contribute to the differential response against *B. cinerea* among the cuticular mutants, RNA transcriptome sequencing (RNA-seq) and analysis were performed on non-infected plants. We identified that the number of differentially expressed genes (DEGs) was different in each mutant, compared with their corresponding WT plant, as follows: 2,386 in *eca2*, 3,506 in *bdg*, 1,688 in *lacs2-3*, 2,819 in *cer1-4*, and 611 in c*er3-6* (Figure 4A and Supplementary Data 1). Additionally, we studied if these DEGs were shared among the mutants (Figure 4B). Interestingly, a large proportion of the DEGs (up- and down-regulated genes) was unique in most of the mutants, except for *lacs2-3* and c*er3-6*. For instance, 68, 51, and 69% of the DEGs were only detected in *eca2*, *bdg*, and *cer1-4*, respectively, while 35 and 23% of the DEGs were only identified in *lacs2-3* and c*er3-6* (Figure 4B). We also looked at DEGs shared only in *B. cinerea*-resistant or susceptible mutants (Supplementary Data 2). The Gene Ontology (GO) analysis of these DEGs from the cutin and wax mutants showed substantial differences. Among the 36 induced genes identified in the three cutin mutants, there is enrichment in the transmembrane receptor protein tyrosine kinase signaling pathway, wax biosynthetic process, and cuticle development. Among the 29 induced genes in the wax mutants, there is enrichment in the regulation of response to stress, regulation of response to external stimulus, and developmental process involved in reproduction (Supplementary Data 3). On the other hand, for 67 genes that are downregulated in the wax mutants, the DEG biological process categories were associated with photosynthesis and response to abiotic stress, while the 33 downregulated genes identified in the cutin mutants were classified as regulation of metabolic process (Supplementary Data 3). Finally, we identified that only two genes were upregulated in all five mutants. They were *GRP3* (AT2G05520), which encodes an Arabidopsis Glycine Rich Protein, and the AT3G14470, which encodes an NB-ARC domain-containing disease resistance protein. Similarly, only two genes were downregulated in all the five mutants: *CBF3* (AT4G25480), which encodes a member of the ERF/AP2 transcription factor family, and a gene that encodes a xyloglucan endotransglycosylase/hydrolase (AT3G44990) (Figure 4B). These results suggest a differential transcriptional regulation in all the cuticular mutants.

### Under Non-challenged Conditions, Cutin Mutants Transcriptionally Induced Differential Defense Responses

**FIGURE 5:**
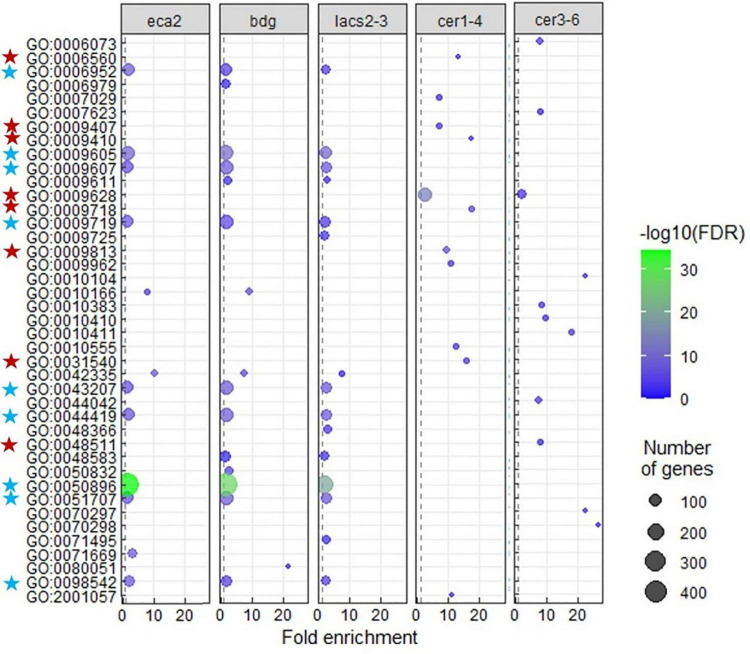
Gene ontology (GO) enrichment among upregulated genes in each cutin or wax mutant. Terms that were largely redundant were removed. Enrichment of “Biological process” GO-terms (FDR < 0.01). The color of the circles indicates the significance of the term (–log FDR), and dot size indicates the number of genes that are associated with that term. The blue stars indicate the GO terms common and mainly related to response to biotic stress, and the red stars indicate the GO terms related to response to abiotic stress, in the cutin and wax mutants, respectively.

A transcriptional modification of plant defense response genes in non-infected cutin mutants has been previously described (Voisin et al., 2009; Nawrath et al., 2013). However, to our knowledge, it has not been shown in wax mutants. The GO analysis on the identified DEGs for each mutant reveals that only *eca2*, *bdg*, and *lacs2-3* show the modification of expression of genes classified as part of the response to biotic stresses (Figure 5 and Supplementary Data 4). These GO processes included: response to biotic stimulus and response to other organisms, involved in interspecies interaction between organisms and defense responses, while in the mutants *cer1-4* and *cer3-6*, statistically significant GO processes were classified into the response to abiotic stresses (Figure 5 and Supplementary Data 4). In order to further characterize these results in cutin mutants, we analyzed the transcriptome profile of selected marker genes related to the jasmonic acid/ethylene- (JA/ET), salicylic acid (SA), and abscisic acid (ABA) pathways that have been described to be induced during the interaction with this necrotrophic pathogen (AbuQamar et al., 2006; Windram et al., 2012) (Supplementary Figure 2, Supplementary Data 1). Interestingly, under non-infected conditions, most of these genes were actually downregulated and only a few were induced. These results suggest that while the basal transcriptomic response to biotic stresses is activated in the cutin mutants, canonical defense responses against this pathogen are not induced before the interaction occurs.

### Plant Defense Response Genes Are Induced Only in Cutin Mutants During the Interaction With the Pathogen

**FIGURE 6:**
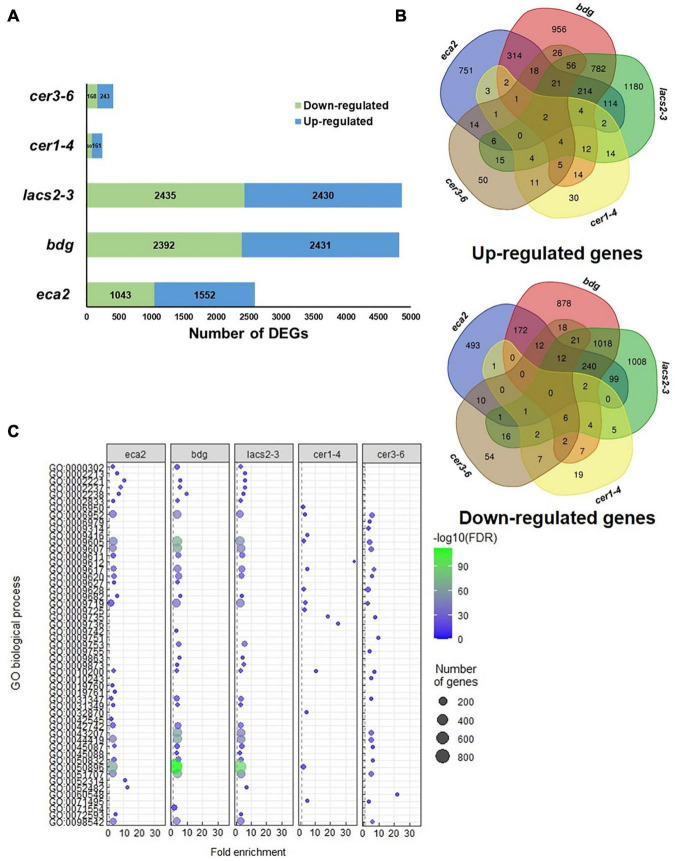
Transcriptome analysis of cuticular mutants infected with *Botrytis cinerea* (6 hpi). **(A)** Number of DEGs (Log2FC ≥ 1 or ≤ –1 with an adjusted *p*-value ≤ 0.05) identified in the cutin and wax mutants. **(B)** Venn diagrams show the overlap of upregulated and downregulated genes among the cutin or wax mutants. **(C)** GO analysis for upregulated genes in each cuticular mutant. Dot plot shows fold enrichment (FDR < 0.01) of the top 25 most significant enrichment terms of biological process.

To further study the modification of the plant transcriptome in cutin and wax mutants, we performed RNA-seq analysis of *B. cinerea-*infected *Arabidopsis* leaves at 6 hpi. Interestingly, we observed a clear difference in the number of genes that are induced or repressed in the cutin mutants compared with the wax mutants. For instance, the number of DEGs in *eca2*, *bdg*, and *lacs2-3* was 2,595, 4,823, and 4,865; while in *cer1-4* and *cer3-6*, the number was only 241 and 411, respectively (Figure 6A, Supplementary Data 5). This represents approximately 10- to 8-fold more DEGs in the cutin mutants than in the mutants with reduced content of wax. To determine the processes that are transcriptionally induced, we performed a GO analysis of upregulated genes (Figure 6C). This analysis reveals a clear enrichment of GO terms related to response to a biotic stimulus only in the mutants with a reduced level of cutin. These terms include the biological process involved in interspecies interaction between organisms, defense response, response to biotic stimulus, response to fungus, and defense response to other organisms, among others (Figure 6C, Supplementary Data 6), while the GO analysis of upregulated genes in *cer1-4* and *cer3-6* reveals enrichment in response to abiotic stimuli, such as response to an organic substance, cytokinin-activated signaling pathway, response to chemical, and response to stimulus (Figure 6C, Supplementary Data 7). Additionally, we found that 214 and 11 DEGs were commonly induced in the cutin and wax mutants, respectively (Figure 6B, Supplementary Data 7). Additionally, 240 common downregulated genes were identified in *eca2*, *bdg*, and *lacs2-3* (Figure 6B, Supplementary Data 7), which were classified into the following GO terms: response to salt stress, response to oxidative stress, response to water deprivation, and response to cold. The seven repressed genes common between *cer3-6* and *cer1-4* belong to response to stimulus, single organism process, and response to external stimulus (Supplementary Data 8). Taken together, these data indicate that the enhanced resistance against *B. cinerea* observed in *eca2*, *bdg*, and *lac2-3* could be explained by the expression of defense-related genes, which are not induced in the wax mutants.

### Canonical Defense Response Genes Are Differentially Induced in Cutin Mutants

**FIGURE 7:**
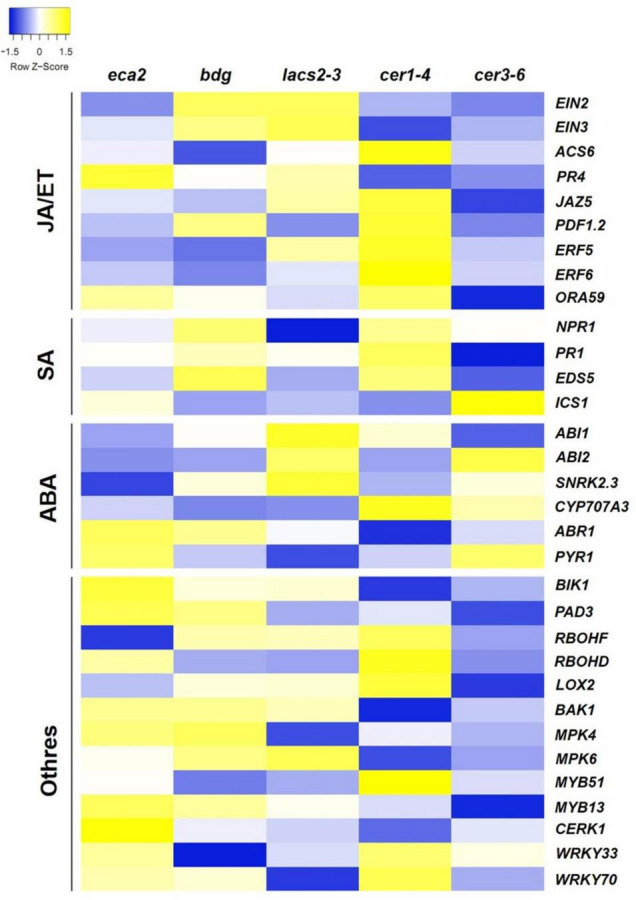
Expression pattern of the jasmonic acid (JA)/ethylene (ET), salicylic acid (SA), and abscisic acid (ABA) signaling pathways and other defense-related genes in response to *B. cinerea* (6 hpi). The colors of the heat map represent the *z*-score of each gene expression calculated between the expression of the mutant compared with its corresponding WT, ranging from blue (–1.5) to yellow (1.5).

We further characterized the defense mechanisms in the mutants with reduced levels of cutin that might participate in the resistance against *B. cinerea*. We were interested in identifying if JA/ET-, SA-, ABA- and/or other defense-related genes were differentially expressed in the cutin and wax mutants (Figure 7). Remarkably, even though many of the hormone- and defense-related genes were induced in all the cutin mutants, we did not detect genes that were expressed simultaneously in all of them, except for the LRR receptor-like protein kinase (*BAK1*) and its interactor *Botrytis*-*induced kinase 1* (*BIK1*), which are involved in the early stages of recognition of pathogens (Veronese et al., 2005; Liu et al., 2017; van der Burgh et al., 2019). This result suggests that resistance against *B. cinerea* might not be exclusively mediated by these well-described genes.

### A Set of Genes Related to Cell Wall Remodeling Is Induced in Mutants With Altered Cutin Monomer Content

**FIGURE 8:**
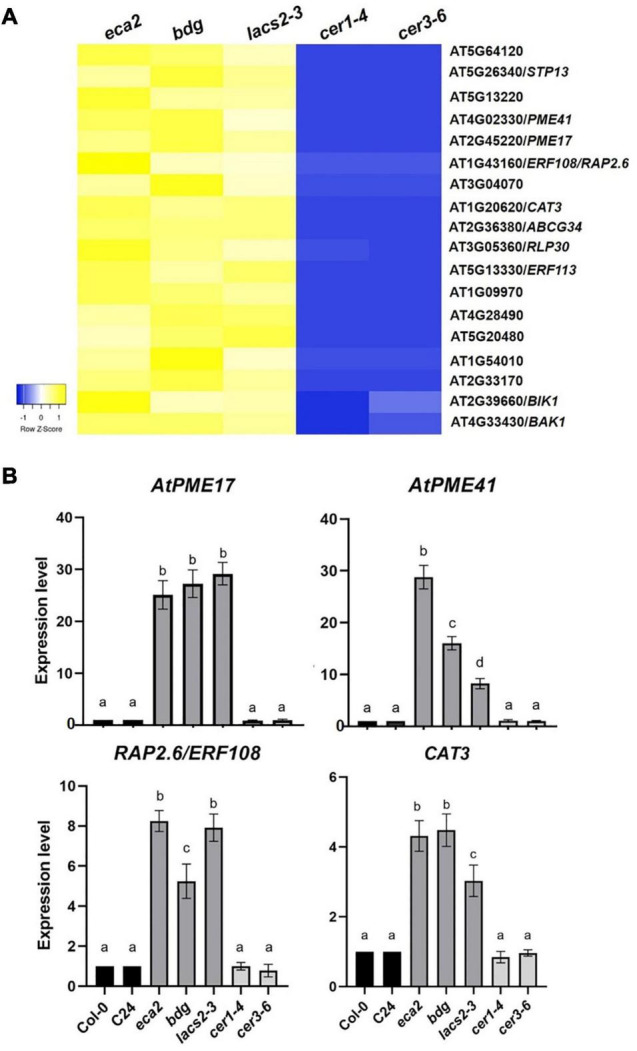
Commonly expressed genes induced in mutants with a reduced level of cutin monomers. **(A)** Heatmap shows the expression levels of upregulated genes shared by the cutin mutants in response to *B. cinerea* (6 hpi). The colors of the heat map represent the z-score of the log2FC of each gene ranging from blue (–1) to yellow (1). **(B)** Expression analysis of some shared up-regulated genes between cutin mutants infected with *B. cinerea*. Expression levels were determined by RT-qPCR in rosette leaves harvested 6 hpi, based on Ct values, normalized with the expression of the two housekeeping genes. The data represent the means of three independent biological replicates with their respective technical replicates, for each sample. Error bars indicate ±SD. Different letters indicate significance differences among samples according to one-way ANOVA followed by Tukey test (*p* ≤ 0.001).

Based on the expression profile of *BAK1* and *BIK1*, we identified all the genes that are commonly upregulated among the three cutin resistant mutants, *eca2*, *bdg*, and *lacs2-3*, but downregulated in *cer1-4* and *cer3-6* (Figures 6B, 8A). We identified 214 genes that share this expression pattern (Supplementary Data 7). From this set of genes, we performed a GO analysis and the genes were classified into the response to biotic stimulus, response to fungus, innate immune response, and defense response to other organisms (Table 1). Interestingly, among these DEGs, we found genes that are involved in cell wall remodelings, such as *AtPME17* (AT2G45220) and *AtPME41* (AT4G02330), and encoding pectin methylesterases (PMEs), which have been recently identified in response to *B. cinerea* (Lionetti et al., 2012; Bellincampi et al., 2014; Bethke et al., 2014, 2016). Likewise, two members of the APETALA2/ETHYLENE RESPONSIVE FACTOR (AP2/ERF) family, transcription factors *RAP2.6/ERF108* and *RAP2.6L/ERF113*, catalase *CAT3*, peroxidases *AtPRX71* [recently identified in resistance to *B. cinerea* (Lorrai et al., 2021)], and Sugar Transporter Protein *STP13*, and genes related to pattern-recognition receptors (PRRs), such as *RLK7*, *RLK5*, and *RLP30*, were identified as DEGs (Figure 8A). In order to confirm these expression patterns and to validate our RNA-seq analysis, we analyzed the expression of *AtPME17*, *AtPME41*, *RAP2.6/ERF108*, and *CAT3* by RT-qPCR (Figure 8B). As expected, we found that all these selected genes were induced 6 hpi in *eca2*, *bdg*, and *lacs2-3*, and that they were downregulated in *cer1-4* and *cer3-6* (Figure 8B).

**TABLE 1:** GO enrichment analysis of Biological process of the 214 common upregulated genes in *eca2*, *bdg* and *lacs2-3* infected with *Botrytis cinerea* (genes are listed in Supplementary Data 6).

**GO_ID**	**GO description**	**Gene** **number**	**Fold** **enrichment**	***p-value***
GO:0002237	response to molecule of bacterial origin	5	18.3	3.76E-02
GO:0045087	innate immune response	9	8.71	4.46E-03
GO:0002376	immune system process	10	6.82	9.56E-03
GO:0010038	response to metal ion	18	6.28	3.62E-06
GO:0009620	response to fungus	12	5.15	1.65E-02
GO:0033993	response to lipid	24	4.44	4.83E-06
GO:0010035	response to inorganic substance	27	4.43	4.33E-07
GO:0098542	defense response to other organism	23	4.28	2.10E-05
GO:0043207	response to external biotic stimulus	29	3.97	1.01E-06
GO:0051707	response to other organism	29	3.97	1.01E-06
GO:0009607	response to biotic stimulus	29	3.97	1.03E-06
GO:0044419	biological process involved in interspecies interaction between organisms	29	3.88	1.70E-06
GO:0006952	defense response	24	3.79	9.29E-05
GO:0009605	response to external stimulus	35	3.47	4.23E-07
GO:0048583	regulation of response to stimulus	17	3.42	4.03E-02
GO:0042221	response to chemical	61	3.41	1.19E-14
GO:0019752	carboxylic acid metabolic process	19	3.38	1.43E-02
GO:1901700	response to oxygen-containing compound	34	3.37	1.72E-06
GO:0006082	organic acid metabolic process	22	3.36	2.72E-03
GO:0043436	oxoacid metabolic process	21	3.33	5.46E-03
GO:0009725	response to hormone	27	3.25	2.58E-04
GO:0009719	response to endogenous stimulus	27	3.18	4.12E-04
GO:0010033	response to organic substance	35	3.07	1.02E-05
GO:0044281	small molecule metabolic process	29	2.92	7.32E-04
GO:0006950	response to stress	59	2.84	1.60E-10
GO:0050896	response to stimulus	91	2.47	1.89E-15
GO:0009987	cellular process	126	1.59	1.69E-08
GO:0008152	metabolic process	84	1.51	3.41E-02

## Discussion

Plants have developed sophisticated responses, such as effects of abiotic and biotic stimuli, to survive in a challenging environment. One of these mechanisms includes preformed physical barriers on the plant surface, such as the cuticle (Chassot et al., 2007; Serrano et al., 2014; Aragón et al., 2017; Bacete et al., 2018; Engelsdorf et al., 2018; Molina et al., 2021). The cuticle, mainly formed by cutin and waxes, also serves as a source of signaling molecules that coordinate the dialog between plants and microorganisms (Aragón et al., 2017; Ziv et al., 2018). In *A. thaliana* and *Solanum lycopersicum*, changes in permeability, associated with modifications in cutin composition, have been linked to resistance against the necrotrophic fungus *B. cinerea* (Bessire et al., 2007; Chassot et al., 2007; Isaacson et al., 2009; Voisin et al., 2009; Martin et al., 2017; Blanc et al., 2018). This resistance has been attributed to faster recognition of pathogens due to a permeable cuticle, accumulation of ROS, and induction of plant defense responses. However, plants with reduced content of waxes, which also have a modified cuticle structure, have not been characterized in detail with respect to this phenotype. In this study, we determined that the wax mutants have a more permeable cuticle but are as susceptible as the wild-type plants. This is in line with the fact that not all cuticle mutants show resistance to *B. cinerea*. For instance, the cuticle mutants *acp4* and *gl1* showed susceptibility to both bacterial pathogens *Pseudomonas syringae* and *B. cinerea* (Xia et al., 2009, 2010; Benikhlef et al., 2013; Lim et al., 2020). Similarly, *shn1*, with cutin monomer content altered, has a more permeable cuticle but is more susceptible to three necrotrophic fungal pathogens, *B. cinerea*, *S. sclerotiorum*, and *Alternaria brassicicola* (Sela et al., 2013; Buxdorf et al., 2014). These results suggest that, as expected, cuticle structural integrity is an important physical barrier against multiple pathogens (such as *B. cinerea*), and that mutants *sma4, lcr*, *bdg, lacs2*, and *eca2*, which possess a low content of cutin, a permeable cuticle, and are resistant to *B. cinerea*, are exceptions to the rule. Additionally, our results indicate that faster recognition of the pathogen by changing cuticle permeability is not enough to induce plant innate immunity responses and resistance to *B. cinerea*, as previously hypothesized (Bessire et al., 2007; Serrano et al., 2014). Nevertheless, these cutin mutants can be used as models to investigate the molecular mechanisms behind the successful induction of defense responses against this agronomically important pathogen.

Plant development and responses to the environment are induced and regulated by signaling networks of “*trio signaling*” messengers: ROS, electrical signals, and calcium (Choi W.G. et al., 2017). In particular, ROS are induced by activating various oxidases and peroxidases in response to abiotic and biotic conditions (Torres and Dangl, 2005; O’Brien et al., 2012; Baxter et al., 2014; Saini et al., 2018). During host-pathogen interactions, the production of ROS is important for plants and for necrotrophic fungi, such as *B. cinerea* and *S. sclerotiorum*. For instance, one of the earliest defense responses to a pathogen attack is the so-called “oxidative burst,” the production of ROS at the site of invasion. A plant releases high amounts of reactive oxygen species (ROS) to counteract the pathogen. On the other hand, *Botrytis cinerea* also takes advantage of this plant defense to kill host cells before they are invaded by hyphae and is able to cope with external oxidative stress in order to survive in the necrotic tissue (Siegmund and Viefhues, 2016). We have described a direct relationship between the accumulation of ROS and resistance to *B. cinerea* after mechanical stimulus or by triggering defense responses through elicitors (Benikhlef et al., 2013; Narváez-Barragán et al., 2020; Batista-Oliveira et al., 2021). Additionally, the resistance against this pathogen observed in the cutin mutants has been also linked to increased levels of ROS under non-infected conditions (reviewed in Serrano et al., 2014). Here, we show that under non-challenged conditions, although all the mutants accumulated more ROS than the WT plants, the cutin mutants *eca2*, *bdg*, and *lacs2-3* have higher levels of superoxide and hydrogen peroxide than the wax mutants *cer1-4* and *cer3-6* (Figure 3). Interestingly, at 6 hpi, all the cuticular mutants have higher levels of superoxide and hydrogen peroxide than the WT plants (Supplementary Figure 1). Despite the *B. cinerea*-induced ROS burst observed in the wax mutants, they showed susceptibility. Two possibilities can be explored to explain this phenotype based on ROS accumulation. First, since a moderate level of ROS is detected in the susceptible mutants *cer1-4* and *cer3-6*, the level of ROS during the initial interaction with the pathogen could be important in triggering resistance against this pathogen. In agreement with this, it has been described that the inhibition of the size of lesions caused by *B. cinerea* is directly proportional to the level of ROS induced by soft mechanical stress (Benikhlef et al., 2013). On the other hand, during biotic interactions, ROS have been characterized to participate in multiple processes, such as reinforcement of the cell wall, regulation of hormone-induced signaling pathways, and triggering of hypersensitive response (Lehmann et al., 2015). Since we observed an increase of ROS in the wax mutants only after the interaction with the pathogen, it is possible that timing, not only the activation of the oxidative burst, is important to efficiently build up a defense response. Either way, our results suggest that besides ROS-dependent resistance, it is probable that other(s) mechanism(s) may be involved to trigger the plant response and, therefore, induce resistance against *B. cinerea*.

To further characterize the molecular mechanisms underlying the resistance or susceptibility in the cuticular mutants, we performed a transcriptomic analysis on the non-infected plants. Up-regulated genes identified in the cutin mutants were classified into the response to biotic stimulus, while GO terms in *cer1-4* and *cer3-6* were mainly associated with response to abiotic stimulus (Figure 5), suggesting that the identified DEGs in cutin mutants could be part of a primed defense response mechanism. These results are in line with previous reports, where a defense priming mechanism has been observed in plants treated with cutin monomers, as well as in other cuticular mutants, leading to expression of a suite of faster and stronger defense responses upon challenge with *B. cinerea* (Schweizer et al., 1996; Fauth et al., 1998; Bessire et al., 2007; Chassot et al., 2007; Conrath, 2011; Conrath et al., 2015; Mauch-Mani et al., 2017). Notably, our data also identified genes related to PRRs, highlighting the leucine-rich repeat receptor kinase (LRR-RK) (Figure 4). Previous studies have reported the importance of PRRs in the priming state. The plant receptor FLS2 and its co-receptor BAK1 were associated with the enhanced responsiveness of *Arabidopsis* plants to the bacterial flagellin peptide flg22 (Tateda et al., 2014), as well as the malectin-like LRR receptor-like kinase IOS1, which is associated with FLS2 and the bacterial receptor EF-Tu (Yeh et al., 2016). Taken together, our results suggest that before the interaction with the pathogen occurs, the cutin mutants are in a priming state compared with the wax mutants or WT plants and that once the infection takes place, they might respond in a faster and more efficient manner, stopping the infection.

Previous reports have characterized secondary responses transcriptionally induced during the interaction with *B. cinerea*. Among these defense responses, SA-, ET-, ABA- and JA-related pathways are induced (AbuQamar et al., 2006; Mengiste, 2012; Windram et al., 2012). To determine if a similar set of genes was present in the cutin and wax mutants, we performed a transcriptome analysis at 6 hpi. In *cer1-4* and *cer3-6* the defense responses are not induced, and most of the DEGs were classified into the response to abiotic stimulus, while in the cutin mutants, enrichment of defense-related genes was identified (Figure 6). However, in *eca2*, *bdg*, and *lacs2-3*, a similar profile of these defense marker genes, which could explain the resistance phenotype for all these mutants based on these hormone-induced responses, was not observed (Figure 7). From this set of genes, only two, involved in the early stages of recognition of pathogen, were identified to be induced in all the cutin mutants: the LRR receptor-like protein kinase (*BAK1*) and its interactor *Botrytis*-*induced kinase 1* (*BIK1*). This result is in accordance with previous reports showing that the *Arabidopsis* mutant *sma4* with a defective cuticle displayed increased resistance to *B. cinerea*, and that this process was independent of the JA and ET signaling pathways (Tang et al., 2007; Wang et al., 2020). Similarly, in CUTE plants, resistance to *B. cinerea* was not found to correlate with the induction of genes associated with the SA, ET, or JA signaling pathways (Chassot et al., 2007). In sum, these results suggest that the resistance to *B. cinerea* observed in *eca2*, *bdg*, and *lacs2-3* might not only be driven by the induction of canonical defense responses, previously identified to be important in stopping the infection.

Since the stronger difference between cutin and wax mutants was resistance and susceptibility to *B. cinerea*, respectively, we hypothesized that a set of genes should be induced only in *eca2*, *bdg*, and *lacs2-3* but repressed in *cer1-4* and *cer3-6* (Supplementary Data 7). Among these DEGs, we identified genes previously characterized to be involved in ROS regulation and cell wall biosynthesis, and are discussed next. The peroxidase (At*PRX71*) and catalases (*CAT3* andAT4G37530) genes were identified as DEGs in the cutin mutants. These genes have been described as part of ROS-scavenging systems to maintain ROS homeostasis in different compartments of the cell, and could, thus, restrict the ROS-dependent damage or finely coordinate the ROS-dependent signal transduction in the presence of a pathogen (Torres et al., 2006; O’Brien et al., 2012). However, necrotrophic pathogens also produce ROS to kill host cells triggering a hypersensitive reaction (HR), thereby facilitating the infection (Govrin and Levine, 2000; Torres et al., 2006; van Kan, 2006). Based on this evidence, it is possible that the expression of these scavengers might help either the plant to inhibit the proper ROS-induced infection process of *B. cinerea*, or regulate ROS-dependent plant defense responses. Clearly, this hypothesis should be tested in future studies.

Additionally, we identified the induction of pectin methylesterases (PMEs) *AtPME41* and *AtPME17* (Figure 8) in the cutin mutants in response to *B. cinerea*. Importantly, these genes have not been reported before as a part of the defense genes against *B. cinerea* in any cuticular mutants. Our data are in accordance with previous results showing that plants activate a local and strong PME activity in response to pathogens with different lifestyles (Lionetti et al., 2012; Bethke et al., 2014; Lionetti, 2015). Once the pathogen overcomes the cuticle, the pectin matrix, which is the major fraction of the cell wall of dicots and non-aminaceous monocots, is the next target for fungal necrotrophs (van Kan, 2006; Laluk and Mengiste, 2010; Lionetti et al., 2012; Bellincampi et al., 2014). PME activity was proposed to be involved in the release and perception of defense of endogenous signals with elicitor activities from cellular components during infection, such as oligogalacturonides (OGs) considered as DAMPs (Lionetti et al., 2012; Ferrari et al., 2013; De Lorenzo et al., 2019). *AtPME41* synthesizes a member of PMEs that has an important role in activating the immune response when *Arabidopsis* is challenged with the necrotroph *A. brassicicola* (Lionetti et al., 2012; Bethke et al., 2014). Additionally, Del Corpo et al. demonstrated the functional role of AtPME17 in triggering PME activity by the JA/ET-dependent pathway and in resistance against pectinolytic necrotrophic fungi, such as *B. cinerea* (Del Corpo et al., 2020).

**FIGURE 9:**
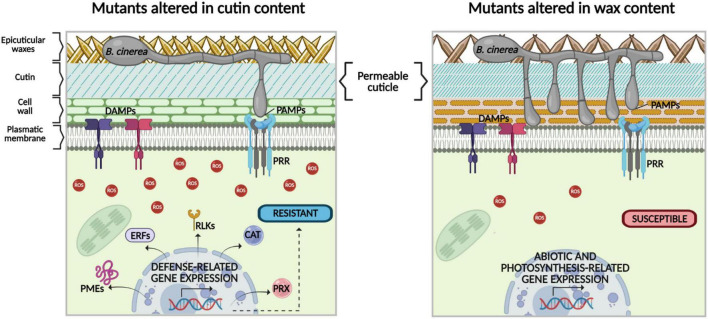
Hypothetical model of the role of cuticular components cutin and wax in differential response to the necrotrophic fungus *B. cinerea*. In previous studies, a direct relationship between increased permeability in cutin mutants and resistance to *B. cinerea* has been proposed. However, our data show that despite both cuticular mutants having enhanced cuticular permeability, compared with the WT plants, only mutants with altered cutin content show resistance to *B. cinerea*. This phenotype could be explained as follows: (1) in the cutin mutants, once plant plasma membrane-localized receptors recognize pathogen-/damage-associated molecular patterns (PAMPs/DAMPs) a more intense ROS production is observed, compared with mutants with altered wax content. (2) During cutin mutant-*B. cinerea* interaction, defense-related signaling pathways are triggered, which are different from canonical immune ones, such as expression genes encoding catalases (*CAT3*) and peroxidases (*PRX*), as well as LRR-receptor-like kinases (*RLK5, RLK7, RLP30*), transcription factors (*ERF108* and *ERF113*), and genes involved in the remodeling of the cell wall, such as *PMEs*. (3) In contrast, according to our data, in the mutants with altered wax content, ROS production is not enough to effectively prevent *B. cinerea* colonization. In these mutants, other secondary signals might be activated to control the expression of genes related to abiotic stimuli, instead of the expression of defense-related genes (the image was designed using bioRENDER application, https://biorender.com/).

Finally, this study illustrates that *B. cinerea* resistance in the cutin mutants *eca2*, *bdg*, and *lacs2-3*, compared with *cer1-4* and *cer3-6*, clearly consists of a multitude of signaling events, from pre-activated defense or priming as initial resistance, production of ROS, and increased expression of non-canonical defense-related genes (Figure 9).

## Conclusion

We have shown that while *cer1-4* and *cer3-6* have altered cuticle permeability, they present a susceptible phenotype, suggesting that the faster recognition of the pathogenic fungus *B. cinerea* is not enough to induce plant innate immune responses and resistance as previously hypothesized. In the cutin mutants *eca2*, *bdg*, and *lacs2-3* responding to *B. cinerea*, a profile of previously characterized defense-response gene markers was not observed, suggesting that mutants with resistant phenotypes can activate other defense pathways, different from these canonical immune ones. Nevertheless, we identified the induction of genes involved in cell wall remodeling in the cutin mutants, which have not been previously reported as part of the defense genes against *B. cinerea* in any cuticular mutants. Based on these results, our study can be used as a starting point to understand the molecular basis involved in early defense mechanisms related to cuticular components against this agronomically important necrotrophic pathogen.

## Data Availability Statement

The datasets presented in this study can be found in online repositories. The names of the repository/repositories and accession number(s) can be found below: NCBI BioProject; PRJNA761130.

## Author Contributions

WA, DF, and MS conceived and designed the experiments. WA, DF, NA-B, and MT performed the experiments. WA, DF, and MS wrote and revised the manuscript. All authors contributed to the article and approved the submitted version.

## Conflict of Interest

The authors declare that the research was conducted in the absence of any commercial or financial relationships that could be construed as a potential conflict of interest.

## Publisher’s Note

All claims expressed in this article are solely those of the authors and do not necessarily represent those of their affiliated organizations, or those of the publisher, the editors and the reviewers. Any product that may be evaluated in this article, or claim that may be made by its manufacturer, is not guaranteed or endorsed by the publisher.

## Footnotes

1
https://imagej.net/Fiji

2
https://www.bgi.com/us

3
https://www.arabidopsis.org/

4
http://bioinformatics.psb.ugent.be/webtools/Venn/

5
http://www.heatmapper.ca/expression/

## Supplementary Material

Supplementary Figure 1 Reactive oxygen species (ROS) production at 6 hpi with *Botrytis cinerea* in leaves from 4-week-old plants of *Arabidopsis thaliana* cuticular mutants and wild-type (WT) Columbia-0 (Col-0) and C24 plants. Fluorescence after DCF-DA staining was observed on leaves by using epifluorescence microscopy. 3, 3′-Diaminobenzidine (DAB) and nitroblue tetrazolium (NBT) were used to detect the accumulation of hydrogen peroxide (H_2_O_2_) and superoxide (O_2_^–^), respectively. The experiment was carried out six times, with similar results (*n* = 6 ± SD). Scale bar = 100 μm. Different lowercase letter columns indicate significant differences, according to one-way analysis of variance (ANOVA) (*p*-value < 0.001) followed by Tukey’s test. Representative pictures are shown.

Supplementary Figure 2 The heat map shows the expression pattern in non-infected cutin and wax mutants, as well as the WT plants of the jasmonic acid (JA)/ethylene (ET), salicylic acid (SA), and abscisic acid (ABA) signaling pathways, and other defense-related genes selected from the literature. The colors of the heat map represent the *z*-score of each gene ranging from blue (−2) to yellow (2).

Supplementary Table 1 Cuticular wax and cutin composition of rosette leaves of Arabidopsis Col-0 (wt), *cer1-4*, *cer3-6* (yre), *bdg*, *lacs2-3*, C24 (wt), and *eca2*.

Supplementary Table 2 Summary of bioinformatics data and mapped reads of *Arabidopsis thaliana* cutin and wax mutants in the RNA-seq libraries in non-infected and infected with *Botrytis cinerea* (6 hpi).

Supplementary Data 1 List of significantly differentially expressed genes (DEGs) in non-infected cutin and wax mutants, related to Figure 4A. List of defense-related genes abundant in the non-infected mutants and wild-type (WT) plants, related to Supplementary Figure 2.

Supplementary Data 2 List of up- and down-regulated genes shared by non-infected cutin or wax mutants, related to Figure 4B.

Supplementary Data 3 Gene Ontology (GO) term enrichment of up- and down-regulated genes shared by non-infected cutin or wax mutants, related to Figure 4B.

Supplementary Data 4 GO term enrichment of up- and downregulated genes in each non-infected cutin or wax mutant, related to Figure 5.

Supplementary Data 5 List of significantly DEGs in cutin and wax mutants infected with *B. cinerea* (6 hpi), related to Figure 6A. List of defense-related genes abundant in the infected mutants and WT plants, related to Figure 7.

Supplementary Data 6 List of up- and downregulated genes shared by cutin or wax mutants infected with *B. cinerea* (6 hpi), related to Figure 6B.

Supplementary Data 7 GO term enrichment of up- and downregulated genes shared by infected cutin or wax mutants, related to Figure 6C.

Supplementary Data 8 GO term enrichment of up- and downregulated genes shared by infected cutin or wax mutants, related to Figure 6B.
