# Supplementary material for: Arabidopsis thaliana Cuticle Composition Contributes to Differential Defense Response to Botrytis cinerea
Source: Front Plant Sci. 2021 Nov 5;12:738949. doi: 10.3389/fpls.2021.738949 (PMC8603936; doi:10.3389/fpls.2021.738949)
Supplement: Supplementary Figure 1 — Reactive oxygen species (ROS) production at 6 hpi with Botrytis cinerea in leaves from 4-week-old plants of Arabidopsis thaliana cuticular mutants and wild-type (WT) Columbia-0 (Col-0) and C24 plants. Fluorescence after DCF-DA staining was observed on leaves by using epifluorescence microscopy. 3, 3′-Diaminobenzidine (DAB) and nitroblue tetrazolium (NBT) were used to detect the accumulation of hydrogen peroxide (H2O2) and superoxide (O2–), respectively. The experiment was carried out six times, with similar results (n = 6 ± SD). Scale bar = 100 μm. Different lowercase letter columns indicate significant differences, according to one-way analysis of variance (ANOVA) (p-value < 0.001) followed by Tukey’s test. Representative pictures are shown. [file Data_Sheet_1.zip › Supplementary Table 1.PDF]

Table S1. Cuticular wax and cutin composition of rosette leaves of Arabidopsis Col-0 (wt), *cer1-4* , *cer3-6* (*yre*), *bdg* , *lacs2-3* , C24 (wt) and *eca2* . Abbreviation: ND, not determined.

Genotype	Total wax loads ($\mu g \cdot dm^{-2}$)	Total cutin loads ($\mu g \cdot dm^{-2}$)	Reference
Col-0 wt	37	ND	Liu et al., 2020
cer1-4	22.13	ND	
cer3-6	14.14	ND	
C24 wt	50	70	Blanc et al., 2018
eca2	23	42	
**Genotype**	**Total wax loads ($\mu g \cdot cm^{-2}$)**	**Total cutin loads ($\mu g \cdot cm^{-2}$)**	**Reference**
Col-0 wt	0.5	1.2	Bessire et al., 2007
lacs2-3	0.4	0.5	
Col-0 wt	0.54	0.64	Kurdyukov et al., 2006
bdg	0.77	1.52	Sadler et al., 2016
