# Supplementary figures and images for: Arabidopsis thaliana Cuticle Composition Contributes to Differential Defense Response to Botrytis cinerea
Source: Front Plant Sci. 2021 Nov 5;12:738949. doi: 10.3389/fpls.2021.738949 (PMC8603936; doi:10.3389/fpls.2021.738949)

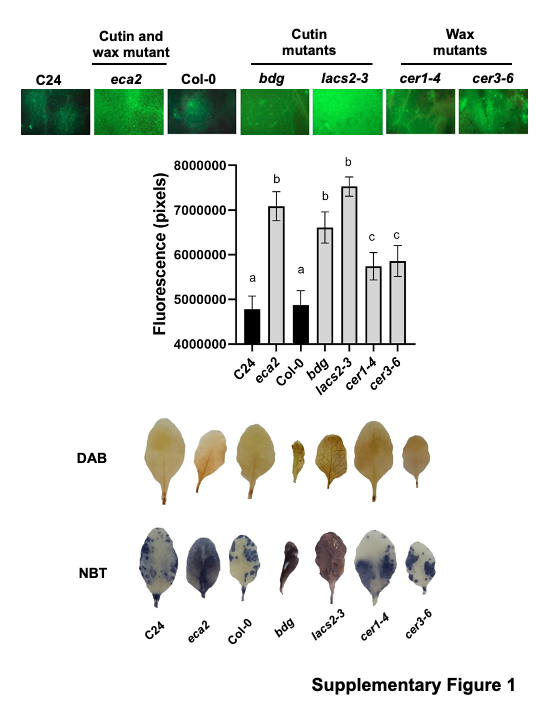

Supplement: Supplementary Figure 1 — Reactive oxygen species (ROS) production at 6 hpi with Botrytis cinerea in leaves from 4-week-old plants of Arabidopsis thaliana cuticular mutants and wild-type (WT) Columbia-0 (Col-0) and C24 plants. Fluorescence after DCF-DA staining was observed on leaves by using epifluorescence microscopy. 3, 3′-Diaminobenzidine (DAB) and nitroblue tetrazolium (NBT) were used to detect the accumulation of hydrogen peroxide (H2O2) and superoxide (O2–), respectively. The experiment was carried out six times, with similar results (n = 6 ± SD). Scale bar = 100 μm. Different lowercase letter columns indicate significant differences, according to one-way analysis of variance (ANOVA) (p-value < 0.001) followed by Tukey’s test. Representative pictures are shown. [file Data_Sheet_1.zip › Supplementary Figure 1.TIF]

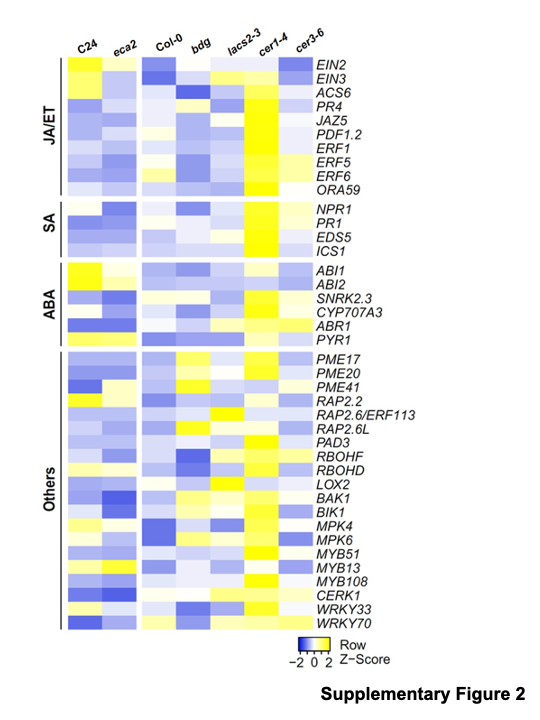

Supplement: Supplementary Figure 1 — Reactive oxygen species (ROS) production at 6 hpi with Botrytis cinerea in leaves from 4-week-old plants of Arabidopsis thaliana cuticular mutants and wild-type (WT) Columbia-0 (Col-0) and C24 plants. Fluorescence after DCF-DA staining was observed on leaves by using epifluorescence microscopy. 3, 3′-Diaminobenzidine (DAB) and nitroblue tetrazolium (NBT) were used to detect the accumulation of hydrogen peroxide (H2O2) and superoxide (O2–), respectively. The experiment was carried out six times, with similar results (n = 6 ± SD). Scale bar = 100 μm. Different lowercase letter columns indicate significant differences, according to one-way analysis of variance (ANOVA) (p-value < 0.001) followed by Tukey’s test. Representative pictures are shown. [file Data_Sheet_1.zip › Supplementary Figure 2.TIF]
